# Carbohydrate Ligands for COVID-19 Spike Proteins

**DOI:** 10.3390/v14020330

**Published:** 2022-02-06

**Authors:** Yung-Kuo Lee, Wen-Chiu Chang, Ekambaranellore Prakash, Yu-Ju Peng, Zhi-Jay Tu, Chun-Hung Lin, Pang-Hung Hsu, Chuan-Fa Chang

**Affiliations:** 1 Department of Medical Laboratory Science and Biotechnology, College of Medicine, National Cheng Kung University, Tainan 70101, Taiwan; yungkuolee@gmail.com; 2 Institute of Basic Medical Science, College of Medicine, National Cheng Kung University, Tainan 70101, Taiwan; 3 Medical Education and Research Center, Kaohsiung Armed Forces General Hospital, No. 2, Zhongzheng 1st Rd., Lingya Distric, Kaohsiung City 80284, Taiwan; 4 Blood Bank, Department of Pathology, National Cheng Kung University Hospital, No. 138, Sheng Li Road, Tainan 704, Taiwan; wenchiu823@gmail.com; 5 Indus Biotech Private Limited, Kondhwa, Pune 411048, India; prakash@indusbiotech.com; 6 Department of Chemistry, National Taiwan University, Taipei 10617, Taiwan; crystalpenginok@gmail.com (Y.-J.P.); chunhung@gate.sinica.edu.tw (C.-H.L.); 7 Institute of Biological Chemistry, Academia Sinica, No. 128 Academia Road Section 2, Nan-Kang, Taipei 11529, Taiwan; s42304@yahoo.com.tw; 8 Genomics Research Center, Academia Sinica, No. 128 Academia Road Section 2, Nan-Kang, Taipei 11529, Taiwan; 9 Department of Bioscience and Biotechnology, National Taiwan Ocean University, No. 2, Beining Rd., Keelung 20224, Taiwan; 10 Center of Excellence for the Oceans, National Taiwan Ocean University, No. 2, Beining Rd., Keelung 20224, Taiwan; 11 Institute of Biochemistry and Molecular Biology, National Yang Ming Chiao Tung University, No. 155, Sec. 2, Linong Street, Taipei 11221, Taiwan

**Keywords:** blood group, carbohydrate ligand, COVID-19, spike protein

## Abstract

An outbreak of SARS-CoV-2 coronavirus (COVID-19) first detected in Wuhan, China, has created a public health emergency all over the world. The pandemic has caused more than 340 million confirmed cases and 5.57 million deaths as of 23 January 2022. Although carbohydrates have been found to play a role in coronavirus binding and infection, the role of cell surface glycans in SARS-CoV-2 infection and pathogenesis is still not understood. Herein, we report that the SARS-CoV-2 spike protein S1 subunit binds specifically to blood group A and B antigens, and that the spike protein S2 subunit has a binding preference for Le^a^ antigens. Further examination of the binding preference for different types of red blood cells (RBCs) indicated that the spike protein S1 subunit preferentially binds with blood group A RBCs, whereas the spike protein S2 subunit prefers to interact with blood group Le^a^ RBCs. Angiotensin converting enzyme 2 (ACE2), a known target of SARS-CoV-2 spike proteins, was identified to be a blood group A antigen-containing glycoprotein. Additionally, 6-sulfo *N*-acetyllactosamine was found to inhibit the binding of the spike protein S1 subunit with blood group A RBCs and reduce the interaction between the spike protein S1 subunit and ACE2.

## 1. Introduction

Since emerging from Wuhan, China in January 2020, the SARS-CoV-2 coronavirus has widely spread around the globe [1]. As of 23 January 2022, more than 210 countries have been affected by the pandemic, 340 million people have been diagnosed with the virus, and more than 5.57 million people have died. Although the number of new cases has been gradually decreasing, daily activities in most parts of the world have remained restricted. Therefore, deciphering the viral infection mechanism is of great urgency to facilitate the control and treatment of COVID-19.

The densely glycosylated spike (S) protein of SARS-CoV-2, a trimeric class I fusion protein with a metastable prefusion conformation [2,3], docks to enter host cells. When binding to a host-cell receptor, the S1 subunit triggers a dramatic structural rearrangement to fuse the viral membrane with the host-cell membrane, leading to receptor-dependent endocytosis [4,5]. These interactions destabilize the prefusion trimer and result in shedding of the S1 subunit and the transition of the S2 subunit into a stable postfusion conformation [6]. Several studies have shown the possible interactions between SARS-CoV-2 or the spike protein with the host receptors (angiotensin-converting enzyme 2 (ACE2) [7,8,9,10]; dipeptidyl peptidase 4 (DPP4) [11,12]; glucose-regulated protein 7 (GRP78) [13,14]); however, the discussion of cell surface glycan receptors used for SARS-CoV-2 viral binding and entry remained vague [15].

Cell surface glycan receptors are known to play a key role in mediating viral binding and infection. Jackson et al. indicated that the entry of foot-and-mouth disease virus (FMDV) into cells is initiated by the contact with heparin sulfate on the cell surface [16]. Sulfated polysaccharides extracted from sea algae have shown potential to prevent the infection of viruses, including herpes simplex virus (HSV), cytomegalovirus (CMV), human immunodeficiency virus (HIV), and enterovirus (EV) [17,18,19,20,21]. Lactoferrin, an 80 kDa iron-binding glycoprotein existing in several mucosal secretions [22,23], has been reported to inhibit the interaction of the capsid protein VP1 of EV71 with rhabdomyosarcoma cells [24,25]. In addition, sialic acids were reported as cell-surface ligands for many viral proteins of influenza virus, parainfluenza virus, reovirus type 3, adenovirus type 37, human rhinovirus 87, human enterovirus type 70 [26], EV-A71 [27], coxsackievirus A24 [28], and hepatitis A virus [29]. Human coronaviruses OC43, HKU1, and MERS were also shown to interact with sialic acids [30,31,32,33]. Clausen et al. indicated that the SARS-CoV-2 spike protein interacts with both cellular heparan sulfate and ACE2 through its receptor-binding domain [34]. Jayaprakash et al. also showed that the S1A domain of the SARS-CoV-2 spike protein may interact with sialosides by molecular modeling [35]. Furthermore, the *N*-glycans of DC-SIGN and L-SIGN that were identified to be the receptors for SARS-CoV-2 could influence the entry of coronavirus [36]. Therefore, it is important to study the role and function of cell surface glycans in the infection of SARS-CoV-2, which appears to be indispensable for understanding viral attachment, infection, and pathogenesis.

We employed a solution carbohydrate microarray to analyze the glycan recognition preference of SARS-CoV-2 spike proteins. The results showed that SARS-CoV-2 spike proteins interacted with several glycans, including blood group antigens, which was coherent with the binding with groups A and B human red blood cells (RBCs). In addition, the effects of carbohydrate analogs in the binding of spike proteins with RBCs were also evaluated.

## 2. Materials and Methods

### 2.1. Glycan Microarray Analysis

Glycan array screening was carried out using a rapid, nonwashing solution carbohydrate array as previously described [37]. Briefly, donor beads (500 ng/well) and biotin-polyacrylamide (PAA)-sugars (20 ng/well) (GlycoTech, Gaithersburg, MD, USA) mixed with SARS-CoV-2 spike protein S1 or S2 subunits (10–20 ng/well, REC31806-100 and REC31807-100 from The Native Antigen Company, Kidlington, Oxford, UK) were incubated at room temperature for 1 h (a total of 15 μL of reaction solution). A mixture of acceptor beads (500 ng/well) and rabbit antisheep IgG Fc antibodies (10 ng/well, ab102297 from Abcam, Cambridge, UK) was added to the reaction to reach a final volume of 25 μL. All reactions were performed in the dark. After incubation for 2 h, the binding signals were measured from a PerkinElmer EnVision instrument and analyzed using the AlphaScreen^TM^ detection program. The results are expressed as relative fluorescence intensities and based on the average from three independent assays.

### 2.2. Binding of Spike Proteins to RBCs Evaluated by Fluorescence-Activated Cell Sorting

The prewashed A, B, and O blood type RBCs were obtained from the Blood Bank of Department of Pathology, National Cheng Kung University Hospital. Two micrograms of spike protein S1 or S2 subunits was incubated with different blood groups of RBCs at 4 °C for 24 h. After incubation, RBCs were isolated from the reaction mixture by centrifugation to remove the nonbinding spike proteins. Rabbit antisheep IgG Fc antibodies (5 μg, purchased from Abcam, ab102297) were added to RBCs and the mixture was incubated at 4 °C for 24 h. RBC samples were then washed three times, followed by the addition of Alexa fluor-488-conjugated goat antirabbit IgG F(ab′)_2_ (2 μg, 23901 from Leadgene, Tainan, Taiwan) and incubated for 1 h at 37 °C. The fluorescence-activated cell sorting (FACS) analysis of spike protein bound RBCs was performed on a FACSCalibur flow cytometer (BD Biosciences). The percentages of blood group A, B, and O RBCs with and without bound spike proteins are indicated in histograms.

### 2.3. Inhibition Assays by Flow Cytometry

Lactose and three sulfated carbohydrate derivatives (20 μg/200 μL) were incubated with spike protein S1 subunits (2 µg) for 12 h and were then mixed with blood group A RBCs at 4 °C for an additional 24 h. After incubation, RBCs were isolated from the reaction mixture by centrifugation then mixed with rabbit anti-sheep Fc antibody (5 μg) at 4 °C for 24 h. Alexa fluor-488-conjugated goat antirabbit IgG F(ab′)_2_ (2 μg) was added after three washes and incubated at 37 °C for 1 h. The percentage of blood group A RBCs with bound spike protein S1 subunits was determined from the FL1 channel in flow cytometry.

### 2.4. Preparation of Lung Tissue Lysate

This study was approved by the National Cheng Kung University Hospital Institutional Review Board (IRB No: A-ER-107-085). The lung cancer tissues were obtained from the Human Biobank of National Cheng Kung University Hospital. The cancer tissues were cut into small pieces and then homogenized thoroughly with the addition of RIPA buffer (Leadgene) and protease inhibitor (Roche). The homogenized tissue samples were centrifuged at 13,000 rpm at 4 °C for 20 min. The supernatant was collected as the whole-cell extract of lung tissue lysate. The protein concentration of the tissue lysate was determined using the Lowry method.

### 2.5. Co-Immunoprecipitation Assays

The lung tissue lysate was precleaned by adding 10 μL of Protein A/G Plus Agarose (Sigma, Merck KGaA, Darmstadt, Germany) and incubated at 4 °C for 1 h. Rabbit anti-ACE2 monoclonal antibody (Proteintech, Chicago, USA), rabbit antiblood group A antibody (ARP), or antirabbit IgG (Genetex, CA, USA) was mixed with Protein A/G Plus Agarose and incubated at 4 °C for 4 h. The antibody–agarose mixtures were individually mixed with precleaned tissue lysate and incubated at 4 °C overnight. After incubation, agaroses were washed 5 times with lysis buffer and the bound proteins were eluted with SDS-PAGE sample buffer.

### 2.6. Western Blotting

The co-immunoprecipitated proteins were mixed with SDS-PAGE sample buffer 6 times, denatured for 5 min at 95 °C, and separated by 6% SDS-PAGE using electrophoresis. Proteins were transferred to PVDF membranes (Millipore, Merck KGaA, Darmstadt, Germany) for 90 min at 300 A and the membranes were blocked for 1 h at 25 °C in 5% milk. Rabbit anti-ACE2 antibody (Proteintech) or mouse antiblood group A antibody (ARP) was incubated with membranes at 4 °C overnight. After being washed 3 times, membranes were incubated with goat antirabbit IgG or goat anti-mouse IgG for 1 h at 25 °C. The detection of the signal was performed with an enhanced chemiluminescence detection kit (Millipore). The gels were digitally photographed and scanned using a gel documentation system (ImageQuant™ LAS 4000).

### 2.7. ELISA

Spike protein S1 subunits (1 μg/mL) were coated on 96-well ELISA plate at 4 °C for 16 h. A blocking procedure was performed by the addition of 0.5% BSA in TBST at 25 °C for 1 h. For inhibition assays, lactose or sulfated carbohydrate derivatives (20 μg/mL) were added into wells and incubated at 25 °C for 1 h followed by two washes. Lung tissue lysates (10 and 1 μg/mL) were added to the well (with and without inhibiter incubation) and incubated at 25 °C for 2 h. After incubation, the reaction mixture in each well was removed and then washed 3 times with PBS. In order to detect the bound ACE2, human ACE2-specific rabbit antibodies (1:1000, Proteintech, Chicago, USA) were added for incubation at 25 °C for 2 h. After washing, HRP-conjugated anti-rabbit IgG antibodies (1:5000, Leadgene) were added for incubation at 37 °C for 1 h. After incubation, the unbound HRP-conjugated antirabbit IgG antibody was washed away and the substrate 3,3′,5,5′-tetramethylbenzidine (TMB, Sigma, St. Louis, MO, USA) was added for incubation at 25 °C for 30 min. Reactions were quenched by adding H_2_SO_4_ (1 N) and the absorbance at 450 nm (OD450) was measured by an ELISA reader (Epoch BioTek) in order to determine the quantity of ACE2 in each well.

## 3. Results

### 3.1. Sugar-Binding Profiling Analysis of SARS-CoV-2 Spike Proteins

**Figure 1:**
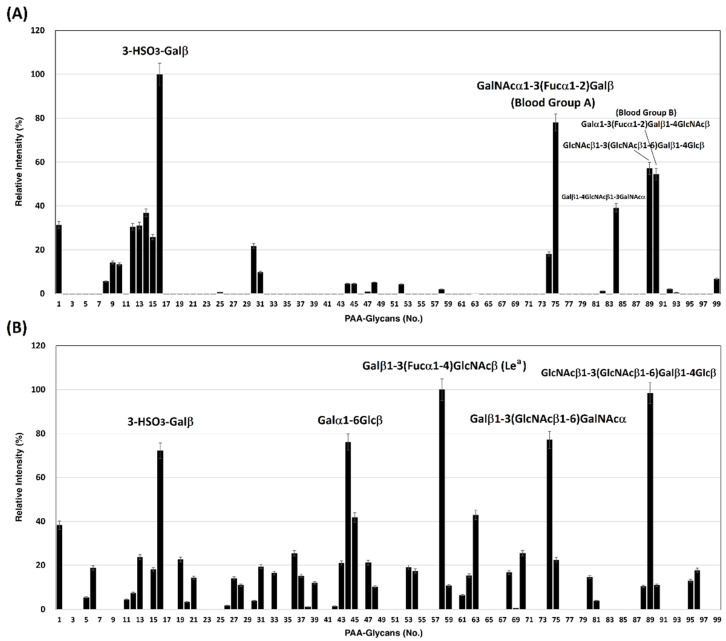
Binding profile of SARS-CoV-2 spike proteins with 97 biotin-PAA-sugars. (**A**) spike protein S1 subunit and (**B**) spike protein S2 subunit.

We previously developed a homogeneous solution carbohydrate microarray in which polyacrylamide-based glycans are used to offer a multivalent environment to screen for specific carbohydrates. There are two advantages to this microarray. This platform can be carried out in a high-throughput manner because the washing step is not required during the screening [37] and is suitable to measure weak binding events that are typical in carbohydrate–protein interactions. So far, this platform has successfully demonstrated the carbohydrate-binding specificities of lectins, antibodies, influenza virus hemagglutinins, and influenza viral particles [38,39]. Using this solution carbohydrate microarray that contains 97 different glycans (Table 1), SARS-CoV-2 spike protein S1 subunits were bound specifically to 3-HSO_3_-Galβ (#16), GalNAcα1-3(Fucα1-2)Galβ (Blood Group A trisaccharide, #75), GlcNAcβ1-3(GlcNAcβ1-6)Galβ1-4Glcβ (#89), and Galα1-3(Fucα1-2)Galβ1-4GlcNAcβ (Blood Group B type 2 tetrasaccharide, #90) (Figure 1A). SARS-CoV-2 spike protein S2 subunits displayed preferential interactions with 3-HSO_3_-Galβ (#16), Galβ1-6Glcβ (melibiose, #44), Galβ1-3(Fucα1-4)GlcNAcβ (Le^a^, #58), Galβ1-3(GlcNAcβ1-6)GalNAcα (#74), and GlcNAcβ1-3(GlcNAcβ1-6)Galβ1-4Glcβ (#89) (Figure 1B). The carbohydrate binding preferences of spike protein S1 and S2 subunits with a relative intensity cutoff of 50% are listed in Table 2.

### 3.2. SARS-CoV-2 Spike Proteins Interact with RBCs

**Figure 2:**
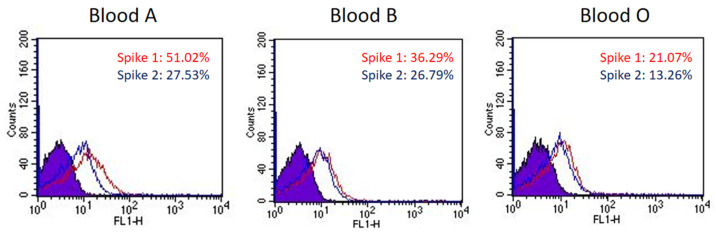
The binding preference of SARS-CoV-2 spike proteins to blood group A, B, and O RBCs. The histograms of spike protein S1 subunits binding with blood group A, B, and O RBCs show a 51.02%, 36.29%, and 21.07% shift in mean fluorescence intensity (MFI), respectively (red line). The histograms of spike protein S2 subunits binding with blood group A, B, and O RBCs show a 27.53%, 26.79%, and 13.26% shift in MFI, respectively (blue line).

The sugar-binding profiling analysis indicated that SARS-CoV-2 spike proteins displayed binding preference for blood-type antigens, including Group A (#75 in Figure 1A), blood Group B (#90 in Figure 1A), and Le^a^ (#58 in Figure 1B). To investigate if the binding preference of SARS-CoV-2 spike proteins correlated with the viral infection or pathogenesis, we examined RBCs that are known to express different blood groups, including group A (Le^a+^/Le^b−^), B (Le^a+^/Le^b−^), and O (Le^a−^/Le^b+^). The binding assay was conducted using fluorescence-activated cell sorting (FACS). The results indicated that the SARS-CoV-2 spike protein S1 subunit binds strongly to group A RBCs, moderately to group B RBCs, and relatively weakly to group O RBCs (Figure 2). The SARS-CoV-2 spike protein S2 subunit displayed higher binding signals with Le^a+^ RBCs than with Le^a−^ RBCs (Figure 2). This observation was consistent with the analysis of carbohydrate microarray, which showed that the spike protein S1 subunit shows a higher preference for blood group A and B RBCs. The binding preference is related to the glycan structures existing on the surface of RBCs.

### 3.3. Carbohydrate Derivatives Interfere with Interaction of SARS-CoV-2 Spike Protein S1 Subunit and Blood Group A RBCs

**Figure 3:**
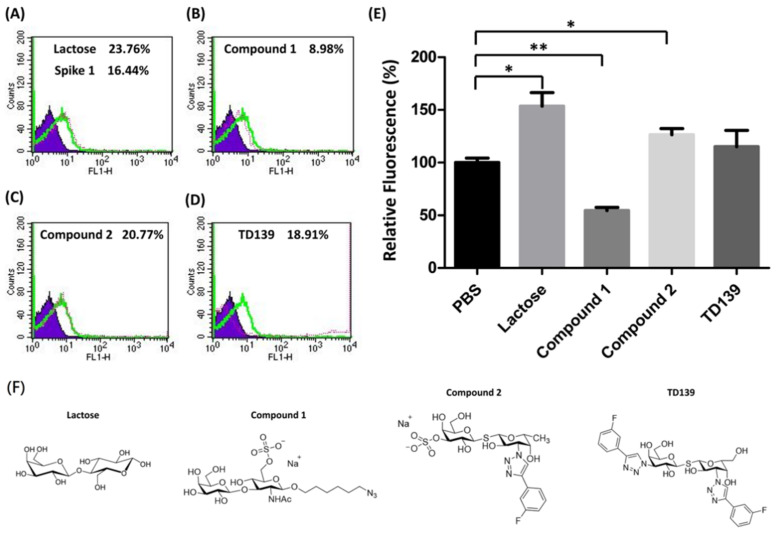
The binding efficiency of the SARS-CoV-2 spike protein S1 subunit to blood group A RBCs affected by carbohydrate derivatives. (**A**) The histogram of spike protein S1 subunit binding with blood group A RBC cells shows a 16.44% shift in MFI without carbohydrate inhibitors. Lactose and three sulfated carbohydrate derivatives were preincubated with spike protein S1 subunit then subjected to the binding assay. The histograms of spike protein S1 subunit binding with blood group A RBC cells are shown (green line). The MFI shifts of blood group A RBC cells with carbohydrate derivatives tested are (**A**) lactose, 23.76%; (**B**) compound **1**, 8.98%; (**C**) compound **2**, 20.77%; and (**D**) TD139, 18.91%. (**E**) The relative fluorescence shows the binding efficiency of the spike protein S1 subunit to blood group A RBCs influenced by carbohydrate derivatives. (**F**) Structures of lactose and three sulfated carbohydrate derivatives. * indicated *p* < 0.05; ** indicated *p* < 0.01.

Carbohydrate analogs are able to interrupt the interaction between microorganisms and host cells by associating with glycoproteins on the surface of either host cells or microorganisms. For example, heparin sulfate mimetics exhibit antiviral activity against dengue virus by inhibiting the virus adsorption on host cells to prevent virus entry [40]. Neuraminidase inhibitors are used for anti-influenza therapy by inhibiting the neuraminidase activity to modify the cell surface glycans, which results in prevention of virions spreading to neighboring cells [41]. Since SARS-CoV-2 spike proteins show a binding preference to blood groups A and B, it is important to examine whether carbohydrate analogs interfere with the interaction between spike proteins and RBCs, especially galactin-3 inhibitors [42]. Lactose and three other carbohydrate derivatives were examined, including compounds **1** and **2** and TD-139. Each of them was preincubated with SARS-CoV-2 spike protein S1 subunit, followed by the addition of blood group A RBCs to the assay mixture (Figure 3A–D). The FACS analysis indicated that compound **1** significantly prevented the binding of spike protein S1 subunit with RBCs up to 45% (Figure 3E, *p* < 0.01). However, lactose and compound **2** enhanced the interaction of spike protein S1 subunit to RBCs by 53% and 26%, respectively (Figure 3E). Interestingly, TD139, a potent inhibitor of galactin-3, exhibited no effect on the spike protein S1 subunit–RBC interaction. The binding inhibition results indicated that carbohydrate analogs containing both sulfate and LacNAc groups reduce the binding affinity between the SARS-CoV-2 spike protein S1 subunit and host cells.

### 3.4. Blood Group A Antigen on ACE2

**Figure 4:**
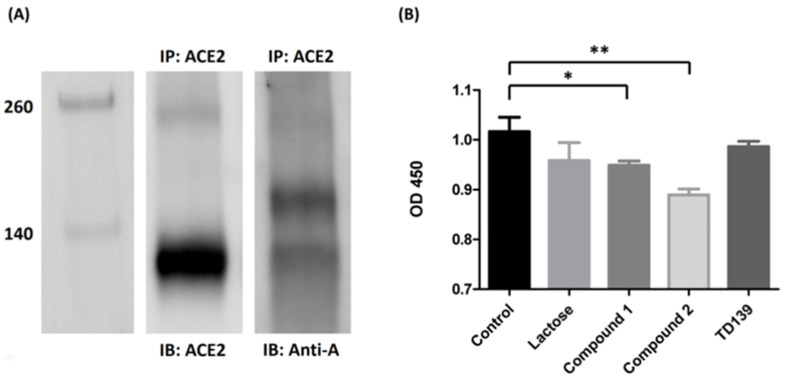
ACE2 contains blood group A antigen. (**A**) Western blotting analysis of ACE2 from the lung tissue of a blood group A patient. Lane 1: markers. Lane 2: the Western blotting of the lung tissue from blood group A patient immunoprecipitated with anti-ACE2 antibody and immunoblotted with anti-ACE2 antibody. Lane 3: the Western blotting of the lung tissue from blood group A patient immunoprecipitated with anti-ACE2 antibody and immunoblotted with anti-A antibody. (**B**) The binding inhibition assay of lactose or sulfated carbohydrate derivatives in blocking of the SARS-CoV-2 spike protein S1 subunit to ACE2. Compounds **1** and **2** showed a reduction in binding efficiency of ACE2 with the spike protein S1 subunit by 6.7% and 12.5%, respectively. TD139 exhibited no effects in spike protein S1 subunit–ACE2 interaction. * indicated *p* < 0.05; ** indicated *p* < 0.01.

Since ACE2 is widely recognized as the major binding target for SARS-CoV-2 spike proteins, it is worth investigating whether the host receptor ACE2 contains the blood group A antigen. ACE2 protein was obtained from the extraction of lung tissues of a blood group A person. The ACE2 protein was immunoprecipitated with anti-ACE2 antibody. Western blotting analysis indicated that the glycoprotein ACE2 in the lung tissue of the blood group A person contained the carbohydrate chains of the blood group A antigen (Figure 4A).

### 3.5. Carbohydrate Derivatives Interfere with Interaction of SARS-CoV-2 Spike Protein S1 Subunit and ACE2

To study if it is possible to disrupt the interaction between SARS-CoV-2 spike protein and ACE2, an ELISA assay was performed to determine the binding inhibition efficiency of carbohydrate derivatives. SARS-CoV-2 spike protein S1 subunits were initially coated on 96-well microplates and incubated with carbohydrate derivatives. After washing away the nonbinding carbohydrate derivatives, human ACE2 proteins prepared from lung tissue lysate by immunoprecipitation were added. The quantities of ACE2 bound on spike protein S1 subunits were determined by ELISA assay. Both compounds **1** and **2** showed a significant decrease in the binding efficiency of ACE2 with the spike protein S1 subunit by 6.7% and 12.5%, respectively (Figure 4B). However, TD139 exhibited no effect on the interaction between spike protein S1 subunit and ACE2. Our results suggested that the specific carbohydrate modifications on ACE2 might be responsible for its binding to SARS-CoV-2 spike protein S1 subunit. Further studies are in progress to decipher the inhibitive effects of these carbohydrate derivatives on the interaction of spike protein S1 subunit and ACE2.

## 4. Discussion

Coronaviruses represent a large family of single-stranded enveloped RNA viruses and can be divided into four major genera [43,44]. Both SARS-CoV and SARS-CoV-2 belong to the β-genus. An envelope-anchored spike protein mediates the entry of the coronavirus into host cells by first binding to a host receptor and then fusing viral and host membranes [4]. A defined receptor-binding domain of the SARS-CoV-2 spike protein was reported to specifically recognize its host receptor ACE2 [7,8,9,10]. The spike protein of SARS-CoV-2 is a glycosylated, trimeric class I fusion protein with a metastable prefusion conformation [45,46]. According to Clausen et al., the SARS-CoV-2 spike protein interacts with both cellular heparan sulfate and ACE2 through its receptor-binding domain [34]. Li et al. showed that the N-terminal domain of β-coronaviridae spike protein S1 subunits (including SARS-CoV2) may potentially interact with unknown glycans [47]. Jayaprakash et al. also indicated that the N-terminal domain of SARS-CoV-2 spike protein binds with sialosides by molecular modeling [35].

ABO blood group antigens have been reported to be associated with diagnosis, prognosis, and survival of various diseases [48,49,50,51,52]. The relationships between ABO blood group antigens and COVID-19 have also been investigated by many researchers. Jawdat et al. found that blood group B is a risk factor for COVID-19 and blood group O is protective factor for COVID-19 infection [53]. Zhao et al. analyzed the ABO blood group distribution among 2173 COVID-19 patients. They found that an increased risk of infection is associated with blood group A and a decreased risk of infection is associated with blood group O [54]. The same findings were also reported in [55,56,57,58,59,60,61]. However, researchers also observed opposite results in other investigations. Zietz et al. showed that non-O blood group types represented slightly increased infection prevalence compared to the O blood group [62]. The results of Ishaq et al. indicated that blood groups have no significant association with severity of COVID-19 disease or COVID-19-associated mortality [63]. Kim et al. also reported that no relationships between blood type and COVID-19-related mortality or severity of illness were observed [64].

Using the solution carbohydrate microarray, we first demonstrated that the SARS-CoV-2 spike protein S1 subunit binds specifically to blood group A and B antigens, and that the SARS-CoV-2 spike protein S2 subunit exhibits a binding preference for Le^a^ antigen (Figure 1). The glycan-binding feature was further investigated by examining how the spike proteins recognize RBCs (Figure 2). Like many C-type lectin domains, the spike protein of SARS-CoV-2 exhibited trimeric fusion protein structure which can enhance protein–glycan interaction by multivalency [65]. Since blood group antigens are available on the surface of RBCs and all human organs/tissues [66] (including saliva [67]), SARS-CoV-2 could easily attach to cell surfaces or droplets and could then be spread and transmitted through the air over time and distance.

Interestingly, COVID-19-induced coagulopathy and a high incidence of thromboembolic events was found in COVID-19-positive deaths [68]. An unknown blood-clotting complication was also reported, even in the patients that were treated with anticoagulants [69]. Unlike Ebola, Dengue, Lassa, and other hemorrhagic fevers that lead to uncontrolled bleeding, SARS-CoV-2 infection was reported to exhibit red, liquid, gel-like blood clots, as well as tiny clots throughout the lungs of dead patients [70]. Our findings may explain why SARS-CoV-2 viral particles potentially agglutinate RBCs by spike–glycan interaction. The proposed unusual agglutination in blood vessels may lead to blood clot formation. Thus, further studies are necessary to demonstrate the correlation and unravel the mechanistic details.

Carbohydrate derivatives such as swainsonine [71,72], 1,4-dideoxy-1,4-imino-D-mannitol [73], deoxymannojirimycin [74,75], and castanospermine [76,77] have shown anticancer activity in different cancer types. Nucleoside analogs are well-developed antiviral drugs for many viral infections including HIV, HBV, dengue virus, yellow fever virus, JEV, and Zika virus [78,79,80]. According to our findings from the solution carbohydrate microarray, the SARS-CoV-2 spike protein S1 subunit preferentially binds to blood group A/B antigens and specific terminal sugar moieties including galactose, *N*-acetylgalactosamine, and sulfated galactose. We evaluated the effects of lactose and sulfated glycan analogs (compounds **1** and **2,** and TD139) on the interaction between the spike protein S1 subunit and RBCs. Compound **1**, a galectin-3 inhibitor, was shown to blockade the interaction of the spike protein S1 subunit with RBCs. However, the inhibition activity was not found in lactose, the other sulfated glycan compound **2**, or TD139. This result suggested that the position of a sulfate group is important, and that sulfated glycans play an important role in the spike protein–glycan interaction.

Since we demonstrated that the SARS-CoV-2 spike protein binds to host cells through interaction with the blood group A antigen and this interaction could be interfered with by glycan analogs, it is worth noting whether the well-known host receptor ACE2 contains the blood type A antigen. ACE2 extracted from the lung tissue of a blood group A patient has shown the expression of the blood type A antigen in Western blotting, indicating that the blood group A antigen is present on ACE2 in the lung tissue of the blood group A person. Though compound **1** showed a significant binding inhibition of spike proteins and RBCs, it poorly inhibited the interaction of the spike protein S1 subunit with ACE2. Compound **2**, however, showed no binding inhibition for spike proteins and RBCs, but displayed better blocking efficiency than compound **1** in the interaction of the spike protein S1 subunit and ACE2. The binding inhibition experiment implied that the interaction of spike protein and RBCs is not only through group A antigen but also involves other groups of blood antigens. The interaction mechanism between these glycan analogs to blood group A antigen or ACE2 needs to be further investigated in the future.

## 5. Conclusions

In conclusion, we identified the carbohydrate ligands for SARS-CoV-2 spike proteins and demonstrated the interactions between SARS-CoV-2 spike proteins with blood group antigens on RBCs. Blood type A antigen serves as one of the possible binding targets of SARS-CoV-2 spike protein S1 subunit, suggesting that blood group A patients may be associated with a higher risk of contracting COVID-19 compared to non-A blood groups. Since we found carbohydrate derivatives to prevent the binding of the SARS-CoV-2 spike protein S1 subunit with RBCs, our results are expected to shed light on SARS-CoV-2 drug discovery.

## Figures and Tables

**Table 1:** List of PAA-glycans.

No.	Glycans	No.	Glycans	No.	Glycans
1	PAA-biotin (backbone)	34	GlcNAcβ1-3Galβ	67	GlcNAcβ1-2Galβ1-3GalNAcα
2	β-GlcNAc	35	Galα1-4GlcNAcβ (αLacNAc)	68	NeuAcα2-3Galβ1-4GlcNAcβ
3	α-Glucose	36	Glcα1-4Glcβ	69	NeuAcα2-3Galβ1-3Glcβn (3’Sialyl Le^c^)
4	β-Glucose	37	Galβ1-3GalNAcα, sp = -p-OC_6_H_4_-	70	Galα1-3Galβ1-4GlcNAcβ, sp = -NHCOCH_2_NH-
5	α-Galactose	38	Galα1-2Galβ	71	GlcNAcα1-3Galβ1-3GalNAcα
6	β-Galactose	39	GlcNAcβ1-4GlcNAcβ	72	NeuAcα2-8NeuAcα2-8NeuAc, (NeuAcα2-8)_3_
7	α-Man-6-phosphate	40	GlcNAcβ1-4GlcNAcβ, sp = -NHCOCH_2_NH-	73	GlcNAcβ1-3Galβ1-3GalNAcα
8	α-L-Rhamnose	41	NeuAcα2-6GalNAcα	74	Galβ1-3(GlcNAcβ1-6)GalNAcα
9	β-GlcNAc	42	3-HSO_3_-Galβ1-4GlcNAcβ	75	GalNAcα1-3(Fucα1-2)Galβ (Blood Group A), sp = (CH2)3NHCO(CH2)5NH-
10	α-GalNAc	43	3-HSO_3_-Galβ1-3GlcNAcβ	76	GlcNAcβ1-3Galβ1-4GlcNAcβ
11	β-GalNAc	44	Galα1-6Glcβ (melibiose)	77	NeuAcα2-3Galβ1-3GalNAcα
12	α-Fuc	45	NeuAcα2-8NeuAcα, (NeuAcα2-8)_2_	78	GlcNAcβ1-3(GlcNAcβ1-6)GalNAcα
13	α-NeuAc	46	Galβ1-2Galβ	79	Galα1-4Galβ1-4GlcNAcβ
14	α-NeuAc-OCH_2_C_6_H_4_-p-NHCOOCH_2_	47	6-HSO_3_-Galβ1-4GlcNAcβ	80	GlcNAcβ1-4GlcNAcβ1-4GlcNAcβ, sp = -NHCOCH2NH-
15	MurNAc-lactic acid-L-Ala-D-isoGln	48	NeuAcα2-3Gal	81	Galβ1-3(NeuAcα2-6)GalNAcα
16	3-HSO_3_-Galβ	49	3-HSO_3_-Galβ1-3GalNAcβ (sulfate-TF)	82	Galβ1-3(NeuAcβ2-6)GalNAcα
17	β-Mannose-PAA-biotin	50	GlcNAcβ1-3GalNAcα	83	NeuAcα2-3(NeuAcα2-6)GalNAcα
18	α-NeuGc	51	GlcNAcβ1-6GalNAcα	84	Galβ1-4GlcNAcβ1-3GalNAcα
19	6-HSO_3_-GlcNAcβ	52	NeuGcα2-6GalNAcα	85	Fucα1-2Galβ1-3(Fucα1-4)GlcNAcβ (Le^b^)
20	GalNAcα1-3Galβ	53	NeuAcβ2-6GalNAcα	86	Fucα1-2Galβ1-4(Fucα1-3)GlcNAcβ (Le^y^)
21	Galα1-3Galβ	54	NeuAcα2-3GalNAcα	87	NeuAcα2-3Galβ1-3(Fucα1-4) GlcNAcβ (sialyl Le^a^)
22	Fucα1-2Galβ	55	GalNAcα1-3(Fucα1-2)Galβ (Blood Group A)	88	NeuAcα2-3Galβ1-4(Fucα1-3) GlcNAcβ (sialyl Le^x^)
23	Galβ1-3GlcNAc (Le^c^)	56	Galα1-3(Fucα1-2)Galβ (Blood Group B)	89	GlcNAcβ1-3(GlcNAcβ1-6)Galβ1-4Glcβ
24	Galβ1-4Glcβ (Lactose)	57	Fucα1-2Galβ1-4GlcNAcβ (H type2)	90	Galα1-3(Fucα1-2)Galβ1-4GlcNAcβ
25	Galβ1-4GlcNAcβ (LacNAc)	58	Galβ1-3(Fucα1-4)GlcNAcβ (Le^a^)	91	Galβ1-3GlcNAcβ1-3Galβ1-4Glcβ
26	Galβ1-3GalNAcα	59	Galβ1-4(Fucα1-3)GlcNAcβ (Le^x^)	92	(NeuAcα2-8)_5-6_
27	Fucα1-3GlcNAcβ	60	Fucα1-2Galβ1-3GlcNAcβ, Le^d^ (H type1)	93	Galβ1-4GlcNAcβ1-3(Galβ1- 4GlcNAcβ1-6)GalNAcα
28	Fucα1-4GlcNAcβ	61	NeuAcα2-3Galβ1-4Glcβ (3’Sialyl Lactose)	94	(NeuAcα2-6Galβ1-4GlcNAcβ1-2Man)_2_α1-3,6Manβ1-4GlcNAcβ1-4GlcNAcβ
29	GalNAcα1-3GalNAcβ	62	NeuAcα2-6Galβ1-4Glcβ (3’Sialyl Lactose)	95	GalNAcα-Ser
30	GalNAcα1-3GalNAcα	63	3-HSO_3_-Galβ1-4(Fucα1-3)GlcNAcβ (3’sulfate Le^x^)	96	GalNAcα1-3(Fucα1-2)Galβ1- 4GlcNAc
31	Galα1-3GalNAcα	64	3-HSO_3_-Galβ1-3(Fucα1-4)GlcNAcβ (3’sulfate Le^a^)	97	Neu5Acα2-3(6-HSO_3_)Galβ1-4(Fucα1-3)GlcNAcβ (6Gal-HSO_3_-SiaLe^x^)
32	Galα1-3GalNAcβ	65	Galα1-4Galβ1-4Glcβ	98	Neu5Acα2-3Galβ1-4(Fucα1-3)(6-HSO_3_)GlcNAcβ (6GlcNAc-HSO_3_- SiaLe^x^)
33	Galβ1-3Galβ	66	Galα1-3Galβ1-4Glcβ	99	H_2_O

Note: sp: spacer.

**Table 2:** The carbohydrate binding preferences of spike protein S1 and S2 subunits (cut off: relative intensity >50%).

SARS-CoV-2 Spike Protein S1 Subunit		SARS-CoV-2 Spike Protein S2 Subunit	
No.	Glycan	No.	Glycan
16	3-HSO_3_-Galβ	16	3-HSO_3_-Galβ
75	GalNAcα1-3(Fucα1-2)Galβ (Blood Group A trisaccharide)	44	Galα1-6Glcβ (melibiose)
89	GlcNAcβ1-3(GlcNAcβ1-6)Galβ1-4Glcβ	58	Galβ1-3(Fucα1-4)GlcNAcβ (Le^a^)
90	Galα1-3(Fucα1-2)Galβ1-4GlcNAcβ (Blood Group B trisaccharide)	74	Galβ1-3(GlcNAcβ1-6)GalNAcα
		89	GlcNAcβ1-3(GlcNAcβ1-6)Galβ1-4Glcβ
